# *Toxoplasma gondii* in a Remote Subsistence Hunting-Based Indigenous Community of the Peruvian Amazon

**DOI:** 10.3390/tropicalmed9050098

**Published:** 2024-04-26

**Authors:** María Fernanda Menajovsky, Johan Espunyes, Gabriela Ulloa, Maritza Calderon, Andrea Diestra, Edith Malaga, Carmen Muñoz, Stephanie Montero, Andres G. Lescano, Meddly L. Santolalla, Oscar Cabezón, Pedro Mayor

**Affiliations:** 1 Departament de Sanitat i Anatomia Animals, Facultat de Veterinària, Universitat Autònoma de Barcelona, 08193 Bellaterra, Spain; mariafernanda.menajovsky@uab.cat; 2 Wildlife Conservation Medicine Research Group (WildCoM), Departament de Medicina i Cirurgia Animals, Universitat Autònoma de Barcelona, 08193 Bellaterra, Spain; johan.espunyes@irta.cat (J.E.); oscar.cabezon@irta.cat (O.C.); 3 Programa de Pós-Graduação em Saúde e Produção Animal na Amazônia, Universidade Federal Rural da Amazônia (UFRA), Belém 66077-830, PA, Brazil; gulloau92@gmail.com; 4 Laboratorio de Investigación en Enfermedades Infecciosas, Universidad Peruana Cayetano Heredia, Lima 15024, Peru; maritza.calderon.s@upch.pe (M.C.); andrea.diestra.c@upch.pe (A.D.); edith.malaga.m@upch.pe (E.M.); 5 Servei de Microbiologia, Hospital de la Santa Creu i Sant Pau, 08041 Barcelona, Spain; cmunoz@santpau.cat; 6 Emerge, Emerging Diseases and Climate Change Research Unit, School of Public Health and Administration, Universidad Peruana Cayetano Heredia, Lima 15015, Peru; stephanie.montero.t@upch.pe (S.M.); willy.lescano@upch.pe (A.G.L.); meddly.santolalla.r@upch.pe (M.L.S.); 7 Clima, Latin American Center of Excellence for Climate Change and Health, and Emerge, Emerging Diseases and Climate Change Research Unit, Universidad Peruana Cayetano Heredia, Lima 15024, Peru; 8 Unitat Mixta d’Investigació IRTA-UAB en Sanitat Animal, Centre de Recerca en Sanitat Animal (CReSA), Campus de la Universitat Autònoma de Barcelona (UAB), 08193 Bellaterra, Spain; 9 ComFauna, Comunidad de Manejo de Fauna Silvestre en la Amazonía y en Latinoamérica, Iquitos 16006, Peru; 10 Museo de Culturas Indígenas Amazónicas, Iquitos 16006, Peru

**Keywords:** *Toxoplasma gondii*, wildlife, wildmeat, bushmeat, indigenous people, One Health, subsistence hunting, Amazon

## Abstract

*Toxoplasma gondii* is a ubiquitous zoonotic protozoan parasite that infects a wide variety range of warm-blooded animals. This study describes the epidemiological scenario of *T. gondii* in an indigenous community that relies on subsistence hunting in a well-conserved and isolated area of the Peruvian Amazon. The high seropositivity against *T. gondii* in humans (83.3% IgG and 6.1% IgM), wild mammals (30.45%, 17 species), peri-domestic rodents (10.0% *Rattus* sp.), and domestic animals (94.1% dogs and 100% cats) indicates the existence of a sylvatic cycle in the community under study. Individual age was found to be positively associated with IgG detection against *T. gondii* but not with IgM. It is estimated that each family consumed 5.67 infected animals per year with terrestrial species having higher infective rates than arboreal species. The main risk factors included improper handling and cooking of wild meat, poor hygiene practices, and feeding uncooked offal to domestic animals. This scenario results in a continuous process of infection and reinfection within the indigenous community with cats, dogs, and peri-domestic animals becoming infected through the ingestion of infected raw viscera. Our results emphasize the need to promote safe food handling practices and disposal of waste materials from hunted animals in such communities.

## 1. Introduction

*Toxoplasma gondii* is an obligate intracellular protozoan parasite that infects a wide variety of wild and domestic warm-blooded animals [1]. This ubiquitous protozoan is one of the world’s most common parasites, infecting an estimated two billion people [2,3]. Felids are the definitive hosts of *T. gondii*, while warm-blooded species (birds and mammals, including humans) act as intermediate hosts and become infected with asexual forms [1]. The most common route of infection is oral, which involves ingesting undercooked contaminated meat, raw vegetables, or water contaminated with oocysts [4,5]. Toxoplasmosis is usually asymptomatic in healthy people, but it can be fatal in young, immunocompromised, or congenitally infected individuals [6,7]. Understanding the factors that contribute to the maintenance and spread of *T. gondii* in the environment is critical for developing effective animal and human health management and prevention strategies [8,9,10].

The Amazon is the world’s largest tropical rainforest with a rich biological and cultural diversity [11]. Amazonian societies rely on a subsistence economy based on hunting and fishing, have limited access to health care, and thus underreport health issues [12]. In addition to close contact with highly biodiverse forested areas in peri-residential environments, these conditions increase the risk of contracting infectious diseases, particularly foodborne pathogens such as *T. gondii* [13,14]. The genetic variability of *T. gondii* in Amazonian countries is very high with several atypical strains causing severe symptoms known as “Amazonian toxoplasmosis” [13]. Furthermore, the recent introduction of cats into Amazonian communities, combined with a lack of safe water sources, increases the risk of *T. gondii* transmission [15]. Toxoplasmosis has rarely been addressed from a One Health perspective in the Amazonian region and remains a neglected disease [1,14,16].

Our study aims to describe the risk of *T. gondii* transmission at the human–wildlife–domestic interface in an isolated community that relies on subsistence hunting in a well-preserved forest in the Peruvian Amazon. This study will improve our understanding of toxoplasmosis by integrating epidemiological data from humans, wildlife, and domestic and peri-domestic animals in the same area. The study area, which is remote, isolated, and well preserved, could provide representative insights into *T. gondii* ecology, transmission dynamics, and infection risk in neotropical forests.

## 2. Materials and Methods

### 2.1. Study Area

**Figure 1:**
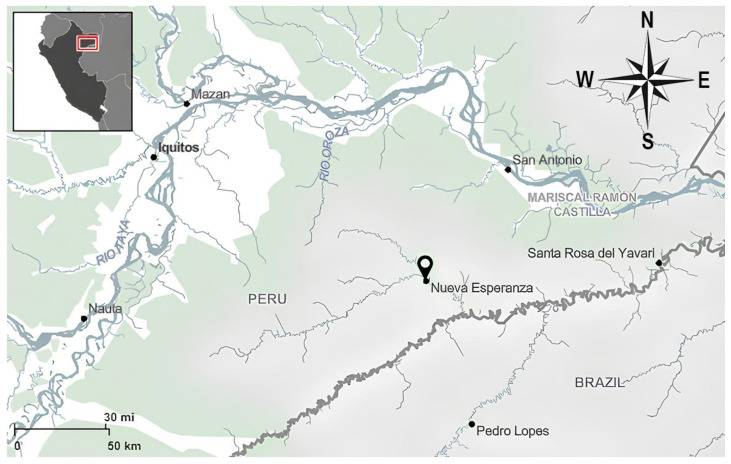
Location of the Nueva Esperanza community in the Yavari-Mirin River basin, a remote area on the border between Peru and Brazil, approximately 150 km far from Iquitos, the closest urban center.

The study was carried out in the Yavari-Mirin basin (04°19′53″ S; 71°57′33″ W; UT5: 00), which is a remote area on the Peru–Brazil border with well-preserved *terra firme* forests in the Peruvian Amazon. The area is rich in biodiversity with up to 150 mammalians and 27 endangered animal species [17,18]. The climate is typically equatorial, with annual temperatures ranging from 22 to 36 °C, relative humidity of 80–100%, and annual rainfall of 1500–3000 mm [19]. The only village still occupied in the Yavari-Mirin River basin is Nueva Esperanza (Figure 1), which is a Yagua indigenous community that had 329 residents and 55 households (with a median of 6 people per household) in 2019.

The main activities of the local community are traditional small-scale agriculture, fishing, logging, and subsistence hunting. Although it is uncommon for residents to travel outside of the village, when they choose to travel, it is usually for short trips to nearby locations, which is primarily motivated by trade-related activities [19]. In 2012, a case of human ocular toxoplasmosis was diagnosed in this community [16].

### 2.2. Blood Samples from Animals and Humans

**Figure 2:**
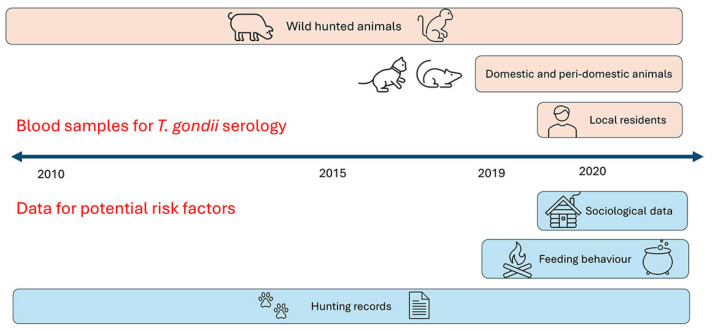
Experimental design diagram showing the chronology of biological sample collection and potential risk factor data collection using semi-structured surveys.

Figure 2 shows the experimental design used, including the chronology of the collection of data and biological samples. Between 2010 and 2020, blood samples were collected from 555 wild mammal individuals belonging to 23 different species, including the orders Primates, Rodentia, Carnivora, Cetartiodactyla, Perissodactyla and Cingulata, as part of a wildlife conservation program, taking advantage of the discarded material from legal subsistence hunting. Hunters impregnated either Protein Saver^®^ or FTA^®^ cards (Scheilcher & Schuell, Dassel, Germany) with blood from the cranial or caudal cava veins. This protocol allows for the obtention and maintenance of blood samples from filter paper, which is a low-cost and technically alternative sampling method in areas where it is difficult to sustain an appropriate cold chain [20]. The sampling was performed in all seasons, and hunters recorded the species, sex, date, and hunting location of each animal. In addition, blood samples from the community’s domestic animals (17 dogs and four cats) and small peri-domestic rodents (40 *Rattus rattus* and five *Mus musculus*) were collected in FTA^®^ cards between September 2019 and February 2020. All FTA cards (wildlife, domestic and peri-domestic fauna) were sealed in individual plastic bags with desiccant and stored at room temperature during the stay in Nueva Esperanza (from 15 to 100 days) before being transferred to −70 °C for preservation.

In February 2020, clinical examination and whole blood collection were performed by physicians on 132 residents (40.1% of the total population): 81 (61.4%) women and 51 (38.6%) men, aged between 4 and 94, with a median age of 21.0 (10.0–34.0). Serum was extracted from the samples and stored in liquid nitrogen for transport and then stored at −70 °C.

### 2.3. Laboratory Procedures

Animal samples were processed by cutting a 132 mm^2^ area of blood-soaked filter paper and diluting it in 400 µL of sterile phosphate-buffered saline (PBS) (Invitrogen, Barcelona, Spain). Considering that the serum accounts for 40% of the total blood volume [21] and the blood concentration on the paper is 40 µL/cm^2^, a serum dilution of 1:20 was obtained. These samples were vortexed for 20 s and then stored at 4 °C for 24 h before being vortexed again for 20 s and frozen at −20 °C until analysis. These elutions were tested for antibodies against *T. gondii* using the ID Screen^®^ Toxoplasmosis IgG Indirect Multispecies ELISA kit (IDvet, Montpellier, France).

For human samples, IgG serostatus against *T. gondii* was determined using an in-house enzyme-linked immunosorbent assay (ELISA) validated with the IBL International^®^ kit [22]. IgM levels were measured using a chemiluminescent microparticle immunoassay (CMIA, Alinity Toxo IgM kit—Abbott). IgM antibodies usually indicate recent exposure to *T. gondii* and first appear 1–2 weeks after infection. IgG antibodies, which indicate past exposure, appear 1 to 3 weeks after IgM, persisting for 12–24 months or even decades, and on occasion are considered lifelong [23].

### 2.4. Social Structure, Hunting and Feeding Characteristics as Potential Risk Factors

In February 2020, family-based interviews were conducted to gain insights into the behavior and dietary habits of the community in order to determine significant risk factors associated with *T. gondii* infection. The semi-structured surveys were divided into sections that addressed different people based on their activities within the community and households. As a result, response rates varied depending on the question. Due to the low individual correspondence between serological results and behavioral factors, no association was made between risk factors and *T. gondii* serology.

#### 2.4.1. Sociological Data

The semi-structured surveys were conducted on 84 heads of families from 42 different households (76.4% of total households). The survey included 47 (55.9%) women and 37 (44.1%) men with a median age of 32 years old (ranging from 18 to 77). Outdoor activities, particularly those related to hunting (number of hunters per household, frequency, animal handling safety measures, and wild meat handling), and human–animal interaction outside and within households (number and species of animals raised at home, presence of cats) were described (Supplementary Table S1). Additionally, field observations related to the human–animal interface, the handling of wild meat, and events related to the health of people living in the community were registered.

#### 2.4.2. Hunting Registers and Preference of Wild Meat Consumption

Between 2010 and 2020, an annual average of 14.3 hunters recorded all hunted prey (median of 11, ranging from 7 to 33). Hunting registers included the number, species, and sex of hunted animals as well as the day and location of the hunt. This information was used to calculate the yearly count of preys hunted and consumed by residents and estimate the consumption of animals infected with *T. gondii* per household considering only the seroprevalences found in species with a minimum of five hunting records. Also, 14 local hunters were surveyed on their prey and the wild meat flavor preferences of all recorded hunted species (n = 25), ranking them on a progressive scale from 1 (highest) to 25 (lowest) preference.

#### 2.4.3. Feeding Behavior

Two surveys were conducted with adult residents in charge of cooking. The first recorded the daily primary dietary intake of seven randomly selected families (12.7% of all households) over 2.43 ± 1.99 months (from January to April 2019, and September to December 2019), totaling of 513 meals. The participants in the second semi-structured survey were 60 adult residents, with 39 (65.0%) women and 21 (35.0%) men, with a median age of 33 years old (ranging from 18 to 73), and the survey included questions about water sources and handling, processing, preservation, and consumption of wild meat (Supplementary Table S2).

### 2.5. Statistical Analyses

#### 2.5.1. Wildlife

Species from all orders with a sample size n > 3 were used to assess the relationship between antibody presence against *T. gondii* using pairwise Fisher’s tests comparisons with Bonferroni correction. A generalized linear mixed model (GLMM) was used to explore relationships between *T. gondii* antibody results in wildlife and their biological and ecological variables. The serological result (negative or positive) was used as a response variable. “Habitat” (terrestrial/arboreal) and “diet” (herbivorous/frugivorous/omnivorous/carnivorous) were used as fixed explanatory variables. Given that the variables “habitat”, “weight” and “diet” are associated with the species rather than the individuals, we supplemented the variable “species” as a random effect.

#### 2.5.2. Humans

A GLM was used to investigate the effect of age and sex on the serological result for IgG and IgM antibodies against *T. gondii*. The serostatus of the individual (positive/negative) was the response variable, whereas the interaction between their age (in years) and their gender (male/female) were the explanatory variables.

Pearson correlation was used to assess whether the mean hunting preference or taste predilection among hunters were correlated with the seroprevalence of the species. For interviews and surveys related to dietary consumption patterns and human behaviors, descriptive statistics were conducted, presenting results as mean ± standard deviation or median and quartile values whenever possible.

All data were analyzed using R 4.2.2 [24]. All significance for hypothesis testing was considered at a Type I error of probability of 0.05.

## 3. Results

### 3.1. Wildlife and Domestic/Peri-Domestic Animals

The overall seroprevalence of *T. gondii* infection in wildlife was 30.45% (169/555; 95% CI 26.8–34.4%). Of the 23 wild species evaluated, we detected at least one seropositive individual in 17, including species from all the analyzed orders: Primates, Rodentia, Cetartiodactyla, Perissodactyla, Carnivora, and Cingulata (Table 1). 

Pairwise Fisher tests did not reveal any significant difference in the seroprevalence among the different taxonomic orders (*p* > 0.05). However, the GLMMs revealed that “habitat” was a statistically predictive value for the prevalence of *T. gondii* (*p* = 0.0285, Estimate ± Std. Error: −0.903 ± 0.41, z value: −2.19). Terrestrial animals presented a higher seroprevalences of antibodies against *T. gondii* compared to arboreal species.

Domestic animals had seroprevalence rates of 94.1% (16/17, 95% CI 73.2%–98.9%) in dogs and 100% (4/4, 95% CI 51–100%) in cats. In peri-domestic animals, *Rattus* sp. presented seroprevalences of 10.0% (4/40, 95% CI 3.9–23.1%), and all *Mus musculus* were negative (0/5, 95% CI 0–43.5%).

### 3.2. Humans

**Figure 3:**
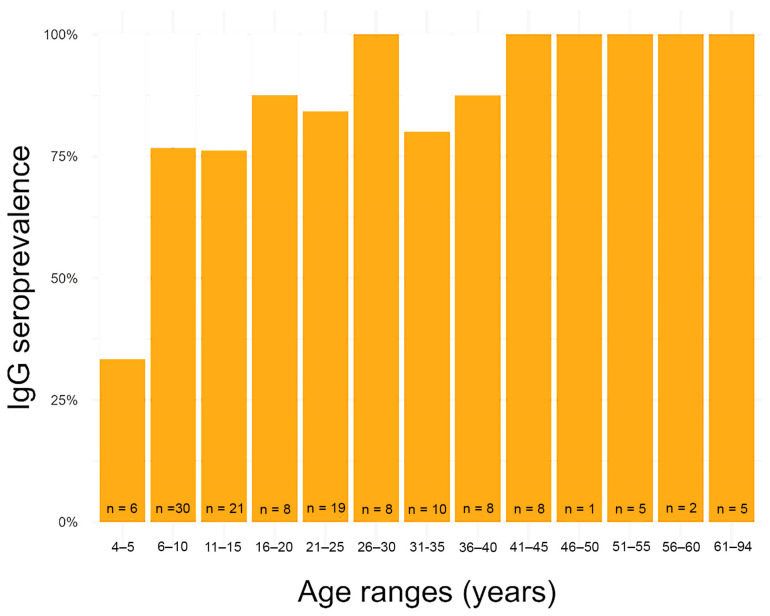
Distribution by age of IgG seroprevalence in residents of the indigenous community (Peruvian Amazon). Blood samples were collected in February 2020.

Overall, 82.6% (109/132, 95% CI 75.2–88.1%) of the Nueva Esperanza residents tested positive to the IgG ELISA, while 6.1% (8/132, 95% CI 3.1–11.5%) tested positive for IgM. All IgM reactive samples also tested positive for IgG. The GLMs indicated that the age of individuals was positively associated with the detection of IgG against *T. gondii* (Estimate ± SE = 0.07 ± 0.03, z value: 2.33, *p* = 0.019; Figure 3) but not with the detection of IgM (*p* > 0.05). The youngest inhabitant with IgG was 5 years old, the average age with IgG was 26 years (26.2 ± 17.7), and the median was 23 years. For IgM antibodies, the average age was 23.5 years old (23.5 ± 20.9), with a median of 10 years old, ranging from 6 to 60 years. There was no significant effect of sex nor the interaction between sex and age (*p* > 0.05) on the serology of IgG nor IgM.

### 3.3. Risk Factors

#### 3.3.1. Sociological Data

Semi-structured surveys revealed that each household had a median of one hunter per household (range from 0 to 2) with a median of hunting frequency of two days per week (range from 0 to 7). A minority (17.7%, 3/17) of the hunters interviewed used dogs to aid in their hunting. None (0%, 0/17) of the surveyed hunters mentioned employing any safety measures (e.g., wearing protective gloves and clothes when handling the hunted animals). The hunters clean the carcass and remove the viscera either in the field (50.0%, 8/16), at home (6.25%, 1/16), or, depending on the animal’s size, in both locations (43.8%, 7/16).

Concerning the interaction with domestic animals, 85.7% (36/42 households) reported having animals at home, including chickens or ducks (81.0%, 34/42 households), dogs (71.4%, 30/42 households), and cats (19.1%, 8/42 households). It was also reported that the first domestic cat was introduced in 2014. Additionally, in 21.4% (9/42) of households, captive wild species as pets were reported, particularly parrots (six households), peccaries (two households) and monkeys (one household). Some households fed their domestic animals with uncooked offal from hunted animals; for instance, dogs and cats (73.8%, 31/42 households) or chickens (59.5%, 25/42 households).

All interviewed heads of families (42/42 households) reported the presence of peri-domestic rodents; 88.1% (37/42 households) reported nests and mouse feces in their house, while 73.8% (31/42 households) mentioned food contaminated with mouse feces. Additionally, identifying rodent sounds at night in the kitchen and food storage was very common, and peri-domestic rodents were captured in all the houses where the traps were placed.

#### 3.3.2. Hunting Records

From 2010 to 2020, local hunters reported a total of 3204 animals, including 2556 (79.8%) mammals from 24 species, 630 (19.7%) birds from 12 species and 18 (0.56%) reptiles from five species.

Considering the serological results for *T. gondii* from our study and the median of one hunter per household, we calculated the estimated number of infected and annually consumed animals per household in Nueva Esperanza. Of the total number of hunted animals registered, it is estimated that 792.8 infected animals were consumed during this 10-year span (Supplementary Table S3). Considering at least one hunter per household, these findings correspond to approximately 14 (25.45%) of the 55 families in Nueva Esperanza. Therefore, it can be inferred that around 5.67 infected animals could have been annually consumed per family. The species exhibiting a higher estimated annual consumption of *T. gondii*-infected meat (individuals/household) were *Cuniculus paca* (n = 2.79), *Pecari tajacu* (n = 0.62), *Tayassu pecari* (n = 0.44), *Lagothrix l. poeppiggi* (n = 0.43), *Mazama americana* (n = 0.41), and *Dasypus novemcinctus* (n = 0.26). None of the variables associated with hunting behavior (prey preference and taste) showed significant associations with *T. gondii* seroprevalence.

#### 3.3.3. Feeding Behaviors

According to the family dietary daily registers, the main source of animal protein was fish, with presence in 50.9% (261/513) of total meals recorded, followed by wild meat (37.8%; 194/513), chicken (9.9%; 51/513), and canned meat (1.4%; 7/513). All wild animals were hunted by men (100.0%, 108/108), whereas fish were predominantly obtained by women (69.3%, 158/228). On a daily basis, each inhabitant consumed 189 ± 139 g (45.5%) of fish, 158 ± 30 g of wild meat (38.0%), and 69 ± 78 g of chicken (16.5%), including non-consumable elements of the meat such as bones, spines, and scales, among others. When asked how they obtained wild meat, mothers from households reported that 60.0% (128/194) of the consumed animals were hunted by the house hunter, 20.6% (40/194) were gifts from relatives or neighbors, and 13.4% (26/194) of the wild animals were purchased.

According to the interviewed inhabitants, the meat was cooked at high temperature (54.8%, 23/42 households), undercooked (11.9%, 5/42 households), or both ways (19.0%, 8/42 households). Offal was consumed by 60.8% (31/51) of local inhabitants, the heart being the most consumed organ (51.0%, 26/51), followed by the liver (47.1%, 24/51), kidneys (15.7%, 8/51), and lungs (9.8%, 5/51). The most frequent method of preserving meat that is not consumed immediately was keeping it fresh (92.9%, 39/42 households), which was followed by drying/salting (66.7%, 28/42 households) and smoking (54.8%, 23/42 households). On the other hand, the water utilized for cooking is predominantly drawn from rivers (28/34, 82.4% households), with rainwater (18/34, 52.9% households) and stream water (2/34, 5.9% households) accounting for smaller proportions. The disposal of wastewater after cooking is carried out in the household yard (76.5%, 26/34 households), in the river (26.5%, 9/34 households), through rustic drainage systems (8.8%, 3/34 households), or in the forest (2.9%, 1/34 households).

## 4. Discussion

Food-borne zoonoses in subsistence-based communities in tropical forests are considered neglected infectious diseases, as evidenced by the scarcity of research studies on this specific wildlife–human interface. Recently, some authors have pointed out the necessity of increasing research efforts into the impact of infectious diseases on public health and wildlife conservation in the Amazon region [25]. The present study evidences the wide circulation of *T. gondii* in wildlife, domestic/peri-domestic animals, and humans in a remote indigenous community of the Peruvian Amazon with a highly interactive human–wildlife interface.

The prevalence of antibodies found in the studied wildlife varied greatly between Orders and was consistent with previous studies from other Amazon areas [26,27] with the exception of the surprisingly low prevalence of infection observed in the Order Carnivora [26,28]. This low sample prevalence in carnivores, particularly wild felids, could be due to the small number of animals sampled, as the local population does not consume them. Specifically, the few wild felines sampled in our study were killed due to conflicts with humans in Nueva Esperanza. Our results indicate that wild terrestrial animals are more exposed to *T. gondii* than arboreal mammals. As felines serve as definitive hosts of the parasite and shed oocysts in their feces, terrestrial species are expected to be infected more frequently [27]. Nonetheless, we also observed high seroprevalences (around 25%) in non-human primates, suggesting that monkeys are also exposed to *T. gondii*. The diets of the primate species studied are primarily frugivorous and supplemented with terrestrial arthropods [29,30]. However, non-human primates occasionally access the ground to obtain nutritional supplements from soils, particularly from mineral licks [31,32], where parasites and diseases spread more widely in wildlife due to high animal density, the variety of species, and the accumulation of feces and urine [33,34].

The serological analyses of domestic and peri-domestic animals showed a high *T. gondii* circulation within the community. The very high prevalence observed in dogs and cats is even higher than that reported in rural communities in the Brazilian Amazon (80% in cats, >50% in dogs) [27,35,36]. Cats are commonly kept outdoors in rural areas, which contributes to *T. gondii* oocyst contamination of the environment [37,38]. In addition, the detection of *T. gondii* antibodies in peri-domestic rodents indicates a wider contamination of immediate household surroundings [39].

The high seroprevalences observed in wildlife and the clinical case of human ocular toxoplasmosis diagnosed in the community [16], both prior to the introduction of cats in the community in 2014, suggest the existence of a sylvatic cycle of the parasite, as previously reported in other regions of Amazonia. However, the emergence of a domestic cat population in the community may have altered the epidemiology of the parasite, making these felines an important player in the maintenance and dissemination of *T. gondii* at the community level, and favoring the establishment of a new domestic cycle of *T. gondii*, as demonstrated in rural areas of Neotropical regions [40]. Therefore, the co-existence of both “wild” and “domestic” cycles may pose an additional risk to human health due to the gene flow between “wild” and “domestic” types of *T. gondii* [40]. Thus, future studies are needed to determine the infective types associated with the “wild” and “domestic” cycles of *T. gondii* in the Amazon.

Risk factors for *T. gondii* infection are usually associated with the traditional lifestyle of indigenous communities, which include activities such as hunting and the consumption of wild meat from an early age [15]. Furthermore, the absence of proper hygienic practices during the handling of wild meat, such as excluding domestic animals from butchery areas, not using personal protective equipment, and not designating surfaces for butchery have been observed in indigenous communities [41]. These practices, combined with cooking practices that involve undercooked or rare meat, as well as the consumption of animal organs, are significant contributors in documented cases of toxoplasmosis in Amazon communities [31,42]. Therefore, the handling and preparation of wild meat is key to the transmission of *T. gondii* in the community and should be addressed in preventive strategies. The present study confirms this previous research, assessing a high IgG seroprevalence in local residents of the community under study. Our findings are also consistent with previous studies where the rate of IgG seroprevalence increases with age, which is probably due to an increased likelihood of exposure to the parasite over time [14,43]. We also found that initial infections occur at a young age, before the age of five, and that the infection rate in children between six and ten can reach 75%. In contrast, there were no significant statistical differences in *T. gondii* IgG seroprevalence between genders, which is probably due to the equal rates of wild meat consumption in men and women [15]. Moreover, the IgM results and the absence of statistical differences between age groups suggest the existence of recurrent infections in the community. The results obtained from the IgG and IgM tests were not confirmed, so we cannot avoid ruling out false positives.

Furthermore, recent studies have highlighted the significance of waterborne transmission of toxoplasmosis caused by the use of unsafe water sources and the consumption of locally grown vegetables [15,37,38,43]. Limited access to running water poses a significant challenge in minimizing food contamination. Residents of rural communities face difficulties in ensuring adequate hygiene, such as washing tools and hands during meat processing as well as properly disposing of wastewater. The observations regarding water access and use in our study present a compelling picture of the challenges faced in maintaining water hygiene and safety. The predominant reliance on natural water sources such as streams, brooks, and wells, without adequate treatment, underscores a significant risk of waterborne diseases.

## 5. Conclusions

The diagnosed human case of *T. gondii* prior to the presence of domestic/feral cats is consistent with a “wild” cycle of *T. gondii* transmission in which individuals are highly exposed to the parasite through the consumption of wildlife meat with the first contact with the parasite occurring in early childhood. However, the high parasite infection rates observed in peri-domestic and domestic animals (including cats) suggest that “wild” and “domestic” types of *T. gondii* may overlap.

Given the biogeographical and cultural context shared with other subsistence hunting communities, and considering that *T. gondii* is possibly the most widespread parasite globally, similar patterns are likely to exist in other Amazonian communities and even in global tropical forests. It is critical that local governments establish strategies to minimize risk factors by guaranteeing access to clean drinking water and enhancing the proper and hygienic handling and consumption of wild meat.

## Figures and Tables

**Table 1:** Occurrence of IgG antibodies against *Toxoplasma gondii* among wild mammals hunted in the indigenous community (Peruvian Amazon) between 2010 and 2020.

Order, Family	Species	Tested	Positive (%)	95% CI
**O. Carnivora**		**22**	**2 (9.1%)**	**2.5–27.8%**
Felidae	*Leopardus pardalis*	1	0 (0.0%)	0.0–79.4%
	*Panthera onca*	2	0 (0.0%)	0.0–65.8%
Procyonidae	*Nasua nasua*	19	2 (10.5%)	2.9–30.4%
**O. Cingulata**		**38**	**17 (44.7%)**	**30.2–60.3%**
Dasypodidae	*Dasypus novemcinctus*	38	17 (44.7%)	30.2–60.3%
**O. Primates**		**155**	**39 (25.2%)**	**19.0–32.5%**
Atelidae	*Alouatta seniculus*	3	1 (33.3%)	6.2–79.2%
	*Ateles chamek*	20	3 (15.0%)	5.2–36.0%
	*Lagothrix l. poeppigii*	66	15 (22.7%)	14.3–34.2%
Pitheciidae	*Cacajao clavus*	16	1 (6.25%)	1.1–28.3%
	*Plecturocebus cupreus*	4	1 (25.0%)	4.6–69.9%
	*Pithecia monachus*	6	0 (0.0%)	0.0–39.0%
Callitrichidae	*Leontocebus fuscicolis*	1	0 (0.0%)	0.0–79.4%
Cebidae	*Cebus albiforns*	7	3 (42.9%)	15.8–75.0%
	*Sapajus macrocephalus*	32	15 (46.9%)	30.9–63.6%
**O. Rodentia**		**148**	**59 (39.9%)**	**32.3–47.9%**
Cuniculidae	*Cuniculus paca*	139	57 (41.0%)	33.2–49.3%
Dasyproctidae	*Dasyprocta fuliginosa*	6	1 (16.7%)	3.0–56.4%
Caviidae	*Galea musteloides*	1	0 (0.0%)	0.0–79.4%
	*Hydrochoerus hydrochaeris*	1	1 (100%)	20.7–100%
Sciuridae	*Sciurus igniventris*	1	0 (0.0%)	0.0–79.4%
**O. Cetartiodactyla**		**171**	**48 (28.1%)**	**21.9–35.2%**
Cervidae	*Mazama americana*	51	13 (25.5%)	15.6–38.9%
	*Mazama nemorivaga*	1	1 (100%)	20.7–100%
Tayassuidae	*Pecari tajacu*	65	18 (27.7%)	18.3–39.6%
	*Tayassu pecari*	54	16 (29.6%)	19.1–42.8%
**O. Perissodactyla**		**21**	**4 (19.1%)**	**7.7–40.0%**
Tapiridae	*Tapirus terrestris*	21	4 (19.1%)	7.7–40.0%
**Total**		**555**	**169 (30.45%)**	**26.8–34.4%**

## Data Availability

The data presented in this study are available on request from the corresponding author. The data are not publicly available because it includes confidential information from people who participated in the study, even if they are anonymous.

## Supplementary Materials

The following supporting information can be downloaded at https://www.mdpi.com/article/10.3390/tropicalmed9050098/s1, Table S1: Semi-structured survey conducted with 84 heads of families, documenting hunting activities and human-animal interactions, in Nueva Esperanza community in the Peruvian Amazon.; Table S2: Semi-structured survey conducted with 60 adult residents, exploring practices related to handling, processing, preservation and consumption of wild meat, in Nueva Esperanza community in the Peruvian Amazon; Table S3: Estimation of *Toxoplasma gondii* infection in wild mammals hunted and recorded in the indigenous community Nueva Esperanza community (Peruvian Amazon) between 2010 and 2020, considering serology found in the present study.
