# Supplementary material for: Toxoplasma gondii in a Remote Subsistence Hunting-Based Indigenous Community of the Peruvian Amazon
Source: Trop Med Infect Dis. 2024 Apr 26;9(5):98. doi: 10.3390/tropicalmed9050098 (PMC11125861; doi:10.3390/tropicalmed9050098)
Supplement: Supplementary file 1 [file tropicalmed-09-00098-s001.zip › tropicalmed-2949593-supplementary.pdf]

Table S1. Semi-structured survey conducted with 84 heads of families, documenting hunting activities and human-animal interactions, in Nueva Esperanza community in the Peruvian Amazon.

RISK FACTORS (SOCIOLOGICAL DATA)		
1	Are you a hunter?	( ) Yes ( ) No
2	How many days a week do you hunt?	Number: ____
3	Do you use any hunting equipment? (including dogs)	( ) Yes ( ) No<br>Which: ______
4	Do you use any safety measures when handling hunted animals?	( ) Yes ( ) No<br>Which: ________
5	Have you noticed any injuries when handling the animals?	( ) Yes ( ) No
6	Do you wash your hands after handling the hunted animals?	( ) Yes ( ) No
7	Do you check if the hunted animals have any injuries?	( ) Yes ( ) No
8	Where do you remove the viscera of the hunted animals?	Place: ___________
9	Where do you dispose of the viscera of the hunted animals?	Place: ___________
10	Do you raise animals at home?	( ) Yes ( ) No
	Cats	( ) Yes ( ) No
	Dogs	( ) Yes ( ) No
	Chickens/ Ducks	( ) Yes ( ) No
	Pigs	( ) Yes ( ) No
	Do you deworm domestic animals?	( ) Yes ( ) No
	Do you deworm vaccine animals?	( ) Yes ( ) No
	Do you have wild animals at home?	( ) Yes ( ) No<br>Which: ________
11	Have any animals died at home recently?	( ) Yes ( ) No
12	Do dogs eat waste organs or meat?	( ) Yes ( ) No
13	Do chickens eat waste organs or meat?	( ) Yes ( ) No
14	Are there any sick animals at home?	( ) Yes ( ) No
15	Presence of mice at home	Place: _________
16	Have you been bitten by mice at home?	( ) Yes ( ) No

17	Have you seen mouse nests or droppings at home?	( ) Yes ( ) No
18	Have you seen food with mouse droppings at home?	( ) Yes ( ) No
19	Presence of bats at home	( ) Yes ( ) No
20	Have you been bitten by bats at home?	( ) Yes ( ) No
21	Do bats sleep at home?	( ) Yes ( ) No
22	Have you seen bat droppings at home?	( ) Yes ( ) No

Table S2. Semi-structured survey conducted with 60 adult residents, exploring practices related to handling, processing, preservation and consumption of wild meat, in Nueva Esperanza community in the Peruvian Amazon.

FEEDING BEHAVIOR		
1	How many days a week do you eat wild game meat?	Number: _____
2	How do you preserve meat that is not consumed?	Fresh ( ) Smoked ( ) Dried/Salted ( )
3	Where do you prepare the meat that is consumed daily?	Home ( ) Outdoors ( )
4	How is the meat prepared in your home?	( )Cooked at low temperature<br>( )Rarely cooked<br>( )Dehydrated with salt (jerky)<br>( )Cooked at high temperature
5	Do you consume meat with injuries?	( ) Yes ( ) No
6	Do you consume animal viscera ? Which ones?	( )Liver ( )Lung<br>( )Kidney ( )Heart<br>( )Fetus ( )Others_________
7	Do you usually eat the viscera of animals that have injuries?	( )Yes, whole( ) No<br>( ) I remove the injuries and consume the healthy part
8	Do you consume animals that are already dead?	( ) Yes ( ) No
9	What water do you use for cooking?	( )Rainwater( )Stream water<br>( )River water( )Treated water<br>( )Other
10	Where do you dispose of wastewater?	Location:_______

Table S3. Estimation of *Toxoplasma gondii* infection in wild mammals hunted and recorded in the indigenous community Nueva Esperanza community (Peruvian Amazon) between 2010 and 2020, considering serology found in the present study.

Species	***T. gondii* seropositivity (%)**	**Number of individuals hunted and registered**	**Estimated number of infected and consumed individuals**
Cuniculus paca	57/139 (41.0%)	952	390.3
Pecari tajacu	18/65 (27.7%)	316	87.5
Tayassu pecari	16/54 (29.6%)	207	61.3
Lagothrix I. poeppiggi	15/66 (22.7%)	268	60.8
Mazama americana	13/51 (25.5%)	227	57.9
Dasypus novemcinctus	17/38 (44.7%)	83	37.1
Sapajus macrocephalus	15/32 (46.9%)	66	30.95
Ateles chamek	3/20 (15.0%)	98	14.7
Tapirus terrestris	4/21 (19.1%)	74	14.1
Cebus albiforns	3/7 (42.9%)	24	10.3
Nasua nasua	2/19 (10.5%)	86	9.0
Dasyprocta fuliginosa	1/6 (16.7%)	34	5.7
Mazama nemorivaga	1/1 (100%)	5	5.0
Plecturocebus cupreus	1/4 (25.0%)	11	2.75
Alouatta seniculus	1/3 (33.3%)	7	2.3
Cacajao clavus	1/16 (6.25%)	33	2.1
Hydrochoerus hydrochaeris	1/1 (100%)	1	1.0
Sciurus cf. igniventris	0/1 (0.0%)	2	0.0
Galea musteloides	0/1 (0.0%)	2	0.0

Leontocebus (Saguinus) fuscicolis	0/1 (0.0%)	28	0.0
Pithecia monachus	0/6 (0.0%)	18	0.0
**Total**		2556	792.8
